# Genetic predisposition of LEPR (rs1137101) gene polymorphism related to type 2 diabetes mellitus – a meta-analysis

**DOI:** 10.1080/07853890.2024.2302520

**Published:** 2024-01-10

**Authors:** Ramakrishnan Veerabathiran, Aswathi P, Iyshwarya BK, Rajasekaran D, Akram Hussain RS

**Affiliations:** a Human Cytogenetics and Genomics Laboratory, Faculty of Allied Health Sciences, Chettinad Hospital and Research Institute, Chettinad Academy of Research and Education, Tamilnadu, India; b Department of General Medicine, Chettinad Hospital and Research Institute, Chettinad Academy of Research and Education, Kelambakkam, Tamilnadu, India

**Keywords:** Type 2 diabetes mellitus, *LEPR* gene, polymorphism, meta-analysis, systematic review

## Abstract

**Background: **Type 2 diabetes mellitus (T2DM) is a multifaceted disease appropriate to elevated blood glucose levels resulting from decreased insulin and beta-cell activity. Using a case–control methodology, researchers have examined the relationship between polymorphisms in LEPR and T2DM in a population from south India.

**Materials and Methods: **We conducted a genetic analysis of 311 participants, and results were accomplished using a case-control study, a meta-analysis of previous studies on LEPR was conducted, and type 2 diabetes genotype distribution across various geographical regions Malaysians, Chinese Han, Kuwait, Iran, Mongolia, and Han Chinese, Greece, Saudi, India (North India, Punjabi), (South India, Tamilnadu). The study involved 254 prospective investigations, and nine association studies were preferred according to preset criteria. Studies were assessed for quality using the Hardy-Weinberg equilibrium (HWE) and the Newcastle–Ottawa Scale (NOS). An analysis of the genetic models was conducted to determine their relationship, statistical analysis was utilized to calculate odds ratios (ORs) and matching 95% confidence intervals (CIs).

**Results: **The *LEPR*-rs1137101 polymorphism in the case-control study was associated with a significant increase in the risk of type 2 diabetes. A meta-analysis revealed a connection between *LEPR* gene polymorphism (rs1137101) and type 2 diabetes risk. Investigators might gain a more profound thought on the significance of the identified genetic variation and its impact on the chance of developing type 2 diabetes by verifying and strengthening previously reported findings. The model of fixed effects was chosen due to the low heterogeneity, and significant associations were observed in the allelic (OR = 0.79, 95% CI [0.70–0.87]), homozygote (OR = 0.58, 95% CI [0.46–0.72]), dominant (OR = 0.66, 95% CI [0.56–0.79]), and recessive (OR = 0.83, 95% CI [0.71–0.96]) genetic models. A Begg’s funnel plot and Egger’s test indicated no publication bias. These findings suggest that the rs1137101 variant in the *LEPR* gene has been linked to a higher risk of T2DM.

**Conclusions: **A larger sample size, however, is required for further research, and consideration of potential confounding factors is needed to validate these associations. Understanding the implications of *LEPR* gene polymorphisms in T2DM susceptibility may contribute to personalized treatment strategies for patients with T2DM.

## Key Messages

- T2DM is the prevailing diabetes type globally, impacting approximately 90% of people living with diabetes. T2DM has become more commonplace, irrespective of socioeconomic status or demographic background.
- T2DM is characterized by hyperglycemia caused by increased blood glucose levels. Recent research indicates a substantial global rise in T2DM cases, suggesting a growing public health concern.
- The development of type 2 diabetes is influenced by both environmental and genetic factors. Among the genetic factors, the *LEPR* gene (leptin receptor gene) has been extensively studied and is associated with insulin sensitivity, hunger regulation, and energy consumption.
- The *LEPR* gene contains a polymorphism known as Gln223Arg, which alters the 223rd amino acid from arginine to glutamine and may influence signal transduction, potentially increasing susceptibility to type 2 diabetes.
- Investigations into the relationship between T2DM susceptibility and the LEPR gene variant (rs1137101) have been carried out through case-control studies and meta-analyses. The results imply that this genetic variation may influence type 2 diabetes risk, particularly in specific ethnic populations. To fully understand the precise mechanisms underlying these associations and to validate them further, more comprehensive research with larger sample sizes is necessary.
- In general, knowledge of the genetic components—such as the variant of the LEPR gene—can offer important insights into the pathophysiology of T2DM and could result in the creation of more specialized and individualized treatment plans.

## 1. Introduction

Diabetic mellitus (DM) is characterized by high blood sugar levels, which can manifest themselves in a variety of forms, such as during fasting, after meals, or in response to oral glucose tolerance tests. The importance of maintaining appropriate blood glucose levels for human health is often underestimated. The development of diabetes is typically linked to the disruption of the pancreas-insulin axis and the improper utilization of insulin [1]. T2DM is the most frequent kind concerning diabetes worldwide, affecting around 90% of individuals with diabetes. There are estimates that 2030 T2DM will affect about 79.4 million people in India, up to 42.3% in China, and 30.8% among Americans. The disease’s increased prevalence is due to the illness [2]. According to International Diabetes Federation statistics, the diabetes prevalence population may rise from 537 million in 2021 to 783 million in 2045. [3]. T2DM, which affects more than 500 million individuals worldwide, has steadily increased over time, regardless of socioeconomic or demographic background [4]. Hyperglycemia, a condition caused by increased blood glucose levels, is the main characteristic of this metabolic illness. Recent research indicates a substantial global rise in T2DM cases [5]. T2DM impacts insulin gene expression and beta cell development in diabetic islets through histone modifications and DNA methylation, critical for modulating mitochondrial genes to regulate diabetes [6].

T2DM development results from genetic variations and environmental influences [7]. Leptin is a peptide hormone produced primarily across white fatty tissue (adipocytes) that regulates insulin sensitivity, hunger, and energy consumption [8–10]. The term ‘leptin’ is derived from the Greek word ‘leptos,’ which means ‘thin,’ indicating its involvement in controlling appetite, food intake, and maintaining energy balance. Leptin interacts with the leptin receptor, an integral membrane protein that belongs to the class I cytokine receptor family inversely found in several organs [11–13]. This interaction significantly affects fat metabolism [14,15]. Sedentary behaviour is a significant factor, and several markers are linked to developing type 2 diabetes [16]. Despite substantial efforts, there is still a long way to go in managing type 2 diabetes since many patients struggle to achieve their treatment goals [17]. When leptin binds to the LEPR receptor in the hypothalamus and adipose tissue, it regulates energy metabolism and maintains the balance of body fat. Leptin is a hormone that is produced by fat cells. Since it plays a role in the development of T2DM, the LEPR gene is therefore frequently referred to as the T2DM gene [18].

The human genes *LEP* and *LEPR* have been extensively studied and characterized, and they are located on chromosomes 7q32.1 and 1p31.3 [19–21]. The *LEPR* gene comprises 20 exons and 19 introns, spanning a length of over 70 kb. It encodes a protein consisting of 1165 amino acids. In human populations, variations and mutations are determined in the *LEPR* gene, primarily within its exons. Among these genetic variations, a polymorphism known as Gln223Arg arises in the 6th exon, where adenine alternated for guanine at position 668. This genetic mutation alters the 223rd amino acid from arginine to glutamine and potentially influences signal transduction, thereby increasing the susceptibility to type 2 diabetes [22]. Numerous research teams conducted genetic association studies on many ethnic communities to study the connection between genetic variants in the *LEPR* gene and type 2 diabetes susceptibility [23]. Single nucleotide polymorphisms (SNPs) are crucial in influencing susceptibility to T2DM. However, inconsistencies were ascertained in the results of these case-control studies, and these discrepancies may be due to various factors, including selection bias, sample size variations, and differences in genotyping techniques. As a result, the rs1137101 polymorphism in the *LEPR* gene was the focus of a comprehensive examination along with meta-analysis employing the ideal Reporting Items for Systematic Reviews and Meta-Analyses (PRISMA) criteria [24]. This study sought to establish whether polymorphisms in the *LEPR* gene, rs1137101, are linked to developing type 2 diabetes among South Indians. The case-control and meta-analysis findings will aid in identifying any possible links between the *LEPR* gene variation and the particular population’s risk of T2DM.

## 2. Methods

### 2.1. Case control

#### 2.1.1. Study subjects

The research was carried out in investigations in the Department of General Medicine at the Chettinad Hospital and Research Institute, Chennai, India. A case-control study focused on 154 individuals diagnosed with T2DM and 157 healthy participants without diabetes or significant illnesses. The healthy participants attended the hospital for routine checkups and were preferred as control subjects. A standardized questionnaire is a series of predetermined inquiries provided to all participants identically, ensuring that the socio-demographic variables data obtained is comparable and trustworthy. An institutional human ethics committee at Chettinad Academy of Research and Education approved the study (225/IHEC/1-19). As part of the consent process, all participants gave informed consent in written form.

The inclusion criteria for this research include men and women with different BMI values and ages. Individuals whose families have a history of T2DM and those who are currently managing their insulin levels are included in the study, as are those with a known duration of T2DM. In contrast, the control group consists of individuals who have no history of T2DM. Conversely, participants who are HIV-positive or pregnant have been excluded from both the case group and the control group. Recent years have seen an increase in incidence, according to the study. The sample size was calculated using the formula below. Checking if the incident from the previous article is pertinent to the current investigation is a crucial part of the process.

N= p(1‐p)(Z/E)2

P = Proportion of the exposed case (Prevalence)

Z = Power value 95% CI (1.96)

E = Margin of Error (0.05)

#### 2.1.2. Blood sample collection for genetic analysis

Using Miller’s technique, genomic DNA was extracted from whole blood [25]. To avoid blood clotting, total blood samples were taken in the amount of 5 ml in tubes coated with EDTA (ethylenediaminetetraacetic acid). The purity and DNA content were determined using UV spectroscopy. ARMS-PCR was employed to ensure the specific genotyping of the selected gene, which is rs1137101. Table 1 provides details about the designed primers.

**Table 1.** Primer and sequence for LEPR gene.

Gene	Primers	Primer Sequence	Allele
LEPR (rs1137101)	Inner Forward -IF	GAAAATCACATCTGGTGGAGTAATTTTACG	A
	Inner Reverse-IR	GGGCTGAACTGACATTAGAGGTGCCT	G
	Outer Forward-OF	AGGCCTGAAGTGTTAGAAGATTCACCTC	Nil
	Outer Reverse-OR	CATTCTAGAAGCCACTCTTAATACCCCCA	

### 2.2. Meta-analysis

#### 2.2.1. Search strategy

**Figure 1.**
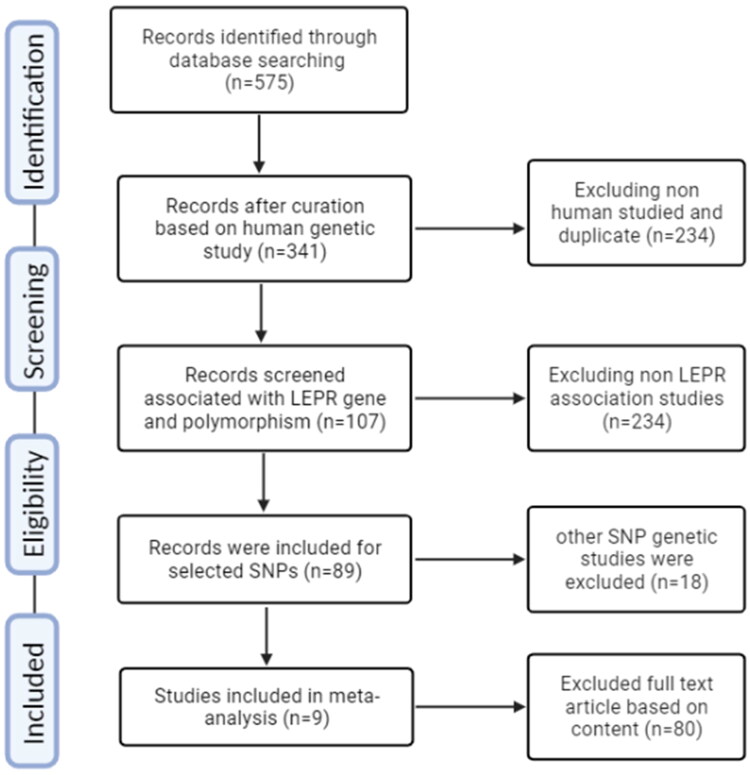
A systematic flow chart detailing the selection of the study.

Many electronic databases were employed to analyze the current literature, including PubMed, Excerpta Medica Database, Cochrane Library, and Google Scholar. The literature search exploration was focused on between 2013 and 2023, and the search terms used were ‘leptin receptor,’ ‘gene,’ ‘lepr,’ ‘T2D,’ and ‘T2DM and Type 2 Diabetes.’ The language was limited to English or Chinese. This study adhered to the guidelines outlined in the PRISMA statement checklist, ensuring accurate and transparent reporting (Figure 1).

#### 2.2.2. Study selection

It was selecting studies that involved adhering to specific criteria for inclusion and exclusion. To be considered, the studies needed to fulfill the subsequent criteria: (1) They had to be case-control studies conducted with human participants, either in hospital settings or utilizing population data, and needed to be original. (2) Type 2 diabetes must be accurately characterized (3) An essential aspect of the study was to assess the connection between the rs1137101 genetic variant and the susceptibility to developing T2D. (4) It was necessary to have ample data to determine the odds ratios of genotypes and alleles and their consistent 95% confidence intervals (CIs). Specific exclusion criteria are exploited to retain this selected study’s exceptional quality and relevance. (1) In our exertion, excluding conference abstracts and retaining accurate editorials and letters would have been possible. (2) Studies conducted on animals or *in vitro* were omitted, as the primary emphasis was on research involving humans. (3) Studies that lacked the necessary genotype frequency data were also excluded. In cases where multiple studies covered the same or similar ground, preference is likely for the study with the most comprehensive information. Furthermore, if numerous studies employed the same pool of persons, the study with the smallest sample size was excluded to prevent duplication and ensure variability among the chosen research.

#### 2.2.3. Data extraction

Three investigators (IBK, RV, and RS) conducted data extraction following a predefined standard protocol. In case of any disagreements, resolve them through discussion. The data extracted from each eligible study consisted of various details, including the publication year, first author’s name, research site, ethnicity, control source (hospital or population-based), type 2 diabetes diagnostic criteria, sample sizes, and genotypic frequencies in both the case and control groups were all recorded.

#### 2.2.4. Methodological quality assessment using HWE and NOS

The HWE and the NOS were employed by the researchers to evaluate the quality of the selected articles [26]. To determine whether the genetic variation at the rs1137101 SNP (single nucleotide polymorphism) within the chosen population complies with the expectations of genetic equilibrium, the Hardy-Weinberg Equilibrium (HWE) analysis was used in this study. This analysis helps ensure the reliability and validity of genetic association studies. The study ensures that genotype distribution complies with HWE; the genotype distribution of the control group should be maintained at a threshold value, generally set at *p* > 0.05 [27]. Three criteria meet the brief for a total potential score of nine points: selection, comparability, and exposure. For this meta-analysis, studies scoring six or higher were deemed acceptable.

#### 2.2.5. Statistical analysis

The goal of this research is to examine the connection between type 2 diabetes risk and polymorphisms in the LEPR gene. The odds ratios are estimated along with 95% confidence intervals (CIs) for different genetic models, including the allelic model (comparing the frequency of P and q alleles), homozygote model (Individuals with two P alleles are correlated to those with two q alleles), heterozygote model (comparing individuals with one P allele to those with two q alleles), dominant model (comparing individuals with one or two P alleles to those with two q alleles), and recessive model (comparing individuals with two P alleles to those with one or two q alleles). The researchers assessed the heterogeneity of the studies using the Thompson and Higgins categorization index (I^2^) and the chi-square-based Q statistic test [28]. Depending on the I^2^ values, they applied either the fixed effects model (Mantel-Haenszel’s method) or the random effects model (DerSimonian and Laird’s method). The random effects model is antiquated if the I2 value is below 50; otherwise, the fixed effects model is employed [29]. They carried out a sensitivity test utilizing the Leave-one-out method, where each study was eliminated one at a time to assess its influence on the final results, to be able to guarantee the reliability of meta-analysis results [30]. Additionally, we evaluated publication bias using funnel plot analysis and Egger’s linear regression test to ascertain whether the publication of particular studies served as an inspiration for the meta-analysis findings [31]. We conducted all statistical analyses using Comprehensive Meta-Analysis software version 2 and STATA version 12.0 (Stata Corp, USA).

**Genotype and Allele Frequency formula**

**Genotype:** Homozygous dominant (AA), Heterozygous (AG), Homozygous recessive (GG).

**Allele:** Allele A (2*AA + AG) and Allele G (2*GG + AG).

## 3. Results

### 3.1. Case-Control study

Based on the demographic data shown in Table 2 for the two groups correlated in this study. We evaluated 311 subjects, consisting of 154 T2DM cases and 157 healthy controls, with a mean age of 44.7 ± 5.4 in cases and 42.08 ± 4.4 in controls. This group included both men and women, but females have been identified at a lower rate than men. The mean body mass index in T2DM patients is 25.2%, while that in controls is 21.1%. A comparison of the two groups revealed that has proven that patients with T2DM have significantly higher body mass index levels than healthy individuals. The study included cases, healthy individuals, and those with a family history of T2DM. There were also documented demographic factors associated with T2DM and controls, including controlling insulin levels, physical activity, eating habits, and diabetes test results. These factors play a significant role in developing diabetes complications.

**Table 2.** Demographical and clinical data of two study groups.

Characteristics	T2DM (*n* = 154)	Controls (*n* = 157)
Men: Women	96:58	83:74
Mean age	44.7 ± 5.45	42.08 ± 4.42
BMI	25.28 ± 2.18	21.53 ± 1.71
Duration of Diabetes	10.16 ± 6.16	Nil
Family History	96	Nil
Controlling insulin level	Tablet:67 Insulin: 87	Nil
Food Habits	Vegetarian: 68 Non-vegetarians: 86	Vegetarian: 99 Non-vegetarians:58
Physical Activity	57	84
Diabetes test	Strip test:48 Lab test: 106	Nil

Data are presented as mean ± SD.

#### 3.1.1. Genetic constitution and allele distribution analysis

**Figure 2.**
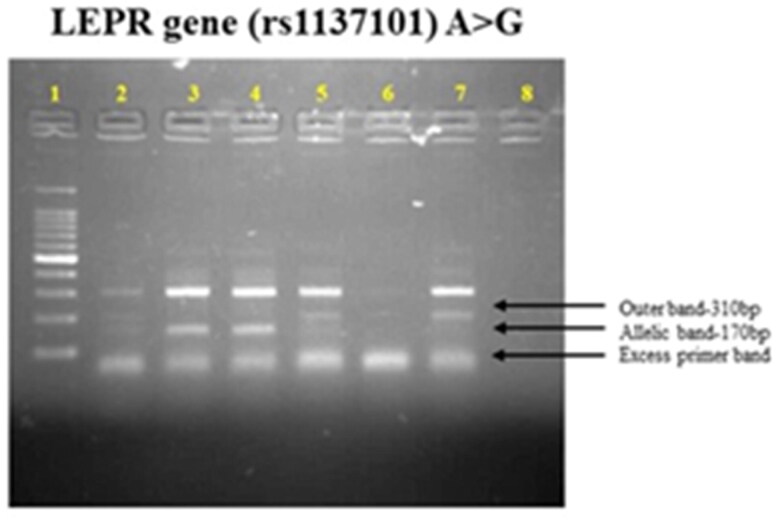
Genotype frequency by ARMS PCR for T2DM and controls.

There was a significant difference in genotypes between T2DM cases (53.24%), AG (15.58%), and GG (31.16%), whereas in control participants (56.05%), AG (24.84%), and GG (19.10%) were detected. The genotype of rs 1137101 is elucidated in Figure 2 of the *LEPR* gene. The derived G allele was shown to be linked to a risk (OR = 0.72, 95% CI-0.51-1.003), with a p-value of 0.03. A risk association between the G allele and type 2 diabetes is indicated by this p-value. Similarly, the homozygous recessive genotype (GG) exhibited a p-value of 0.004 (OR = 0.38, 95% CI-0.19–0.76). In contrast with age-matched controls, patients with T2DM have had a significantly increased risk of developing the rs1137101 polymorphism (*p* < 0.05) (Table 3). The current study results are consistent with many global studies that have reported an association between the LEPR-rs1137101 polymorphism and an increased risk of T2DM. According to meta-analysis research, the LEPR-rs1137101 polymorphism is linked to an elevated risk of T2DM in numerous populations throughout the globe [32].

**Table 3.** Allele frequencies and genotype distribution of LEPR (rs1137101*)* gene polymorphism in T2DM and controls.

Models	Case *N* = 154 (%)	Control *N* = 157 (%)	Odds Ratio 95% CI	*P* value
Genotype				
AA	82 (53.24)	88 (56.05)	1.51 (0.83-2.7)	0.10
AG	24 (15.58)	39 (24.84)		
GG	48 (31.16)	30 (19.10)	0.38 (0.19-0.76)	0.004
Allele				
A	188 (61.03)	215 (68.47)	0.72 (0.51-1.003)	0.03*
G	120 (38.96)	99 (31.5)		

### 3.2. Meta-analysis

#### 3.2.1. Characteristics of the study

In a case-control meta-analysis, a systematic approach was followed to select and review relevant publications. The process involved adhering to the PRISMA guidelines, it took place in phases. Initially, a comprehensive search yielded a pool of 575 publications related to the specific genetic variant under investigation. These publications were collected and subsequently screened to determine their relevance to the research topic. A set of predefined inclusion and exclusion criteria was meticulously applied to the collected publications. These standards were created to separate out studies that did not fit the study’s focus from those that did and to find studies that satisfied the goals of the research. Following the initial screening, a rigorous quality assessment was performed on the selected publications. Two critical criteria were evaluated: (a) adherence to the HWE and (b) the NOS score [33–40], which assesses the quality of observational studies. This step ensured that the chosen studies met the minimum quality standards for inclusion in the meta-analysis. Nine publications in total were found to meet the predetermined quality criteria after a thorough evaluation, making them eligible for inclusion in the next meta-analysis. These studies were selected based on their ability to contribute valuable data to the research. In Table 4, each of the nine selected studies was described based on their unique characteristics, including the NOS score, which provides insights into the quality of each study. This comprehensive characterization allows for a clear understanding of the source and nature of the data being used in the meta-analysis. In Table 5, essential data related to the genetic variant of interest was compiled for the selected studies. This data includes genotype distribution, allelic frequency, and HWE/Chi-square values, which are critical for assessing the consistency and validity of the genetic data across the studies. The comprehensive approach to literature selection and quality assessment ensures that the upcoming meta-analysis will be based on a robust foundation of reliable and relevant studies. By conducting a meta-analysis with these carefully chosen publications, the research aims to draw more powerful and generalizable conclusions regarding the genetic variant’s association with the condition under investigation while minimizing the potential for plagiarism by presenting the findings clearly and originally.

**Table 4.** Characteristics of the study included in meta-analysis.

Study Name	Year	Country	Source of DNA	No of Cases	No of Control	NOS Score	Genotyping Method
Ali Etemad et al. ^[^33^]^	2013	Malaysia	Buccal Specimen	300	299	6	PCR-RFLP
Bo Jiang et al. [34]	2014	Chinese Han	Blood	177	153	7	–
Fatmah Lari et al. [35]	2022	Kuwait	Blood	203	162	8	Taqman genotyping assay
Ghorban et al. [36]	2013	Iran	Blood	144	147	8	PCR-RFLP
Ke-Yu Zhao et al. [37]	2022	Han Chinese	Blood	486	455	8	Multiplex PCR
Maria Trapali et al. [38]	2021	Greek	Blood	108	52	7	PCR-RFLP
Rowyda, et al. [39]	2013	Saudi	Blood	150	130	7	PCR-RFLP
This study	2019	South India	Blood	154	157	8	ARMS-PCR
Veena Bains et al. [40]	2020	Indian	Blood	417	400	7	PCR-RFLP

**Table 5.** Genotyping and allele frequency of LEPR (rs1137101*)* gene polymorphism.

Author	Genotype						Allele				X2	HWE
	Case			Control			Case		Control			
	GG	GA	AA	GG	GA	AA	G	A	G	A		
Ali Etemad et al. [33]	96	20	184	55	41	203	212	388	151	447	121.23	0
Bo Jiang et al. [34]	3	34	140	3	33	117	40	314	39	267	0.14	0.7
Fatmah Lari et al. [35]	18	74	111	15	56	91	110	296	86	238	2.08	0.14
Ghorban et al. [36]	5	59	80	5	62	80	69	219	71	223	2.89	0.08
Ke-Yu Zhao et al. [37]	394	84	8	351	94	10	872	100	796	114	1.99	0.15
Maria Trapali et al. [38]	14	48	46	4	14	34	35	64	21	78	1.93	0.16
Rowyda et al. [39]	92	50	8	78	40	12	234	66	196	64	3.79	0.05
This study	48	24	82	30	39	88	120	188	90	215	28.3	0
Veena Bains et al. [40]	55	177	185	28	172	200	287	547	228	572	1.21	0.27

#### 3.2.2. Association between rs1137101 polymorphism also T2DM vulnerability

**Figure 3.**
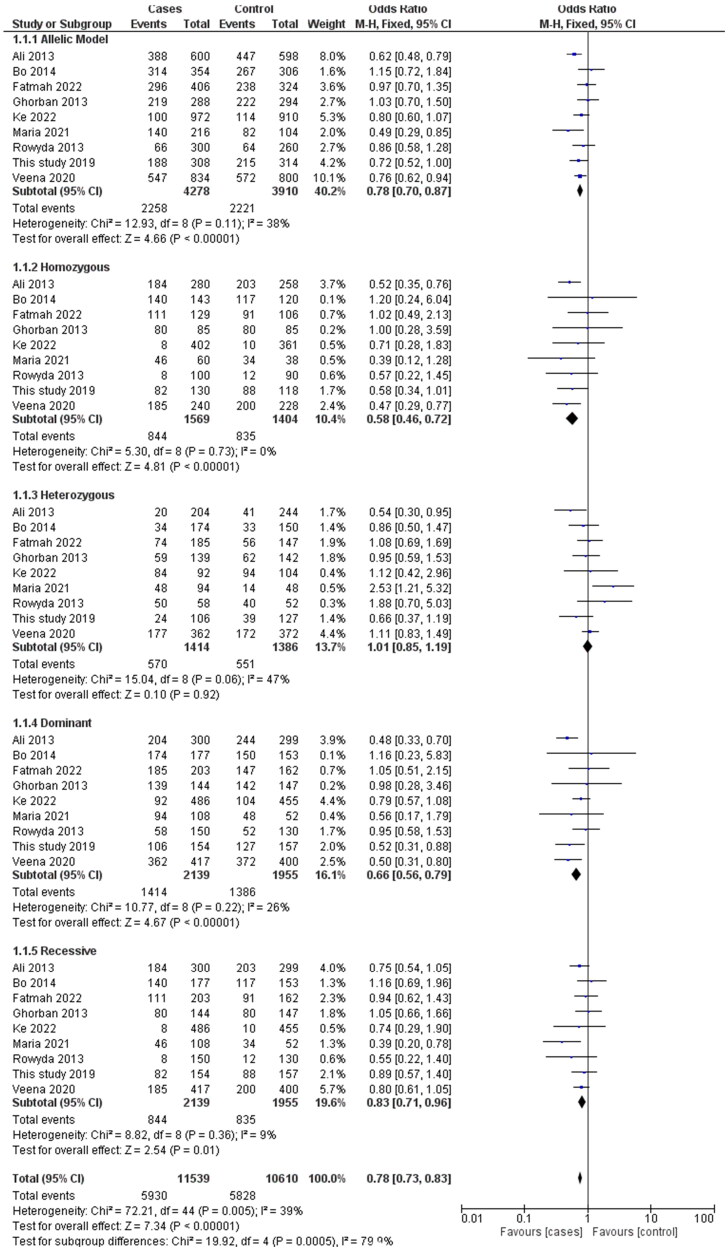
Forest Plot shows the association between the LEPR gene in allele and genotyping frequency.

**Figure 4.**
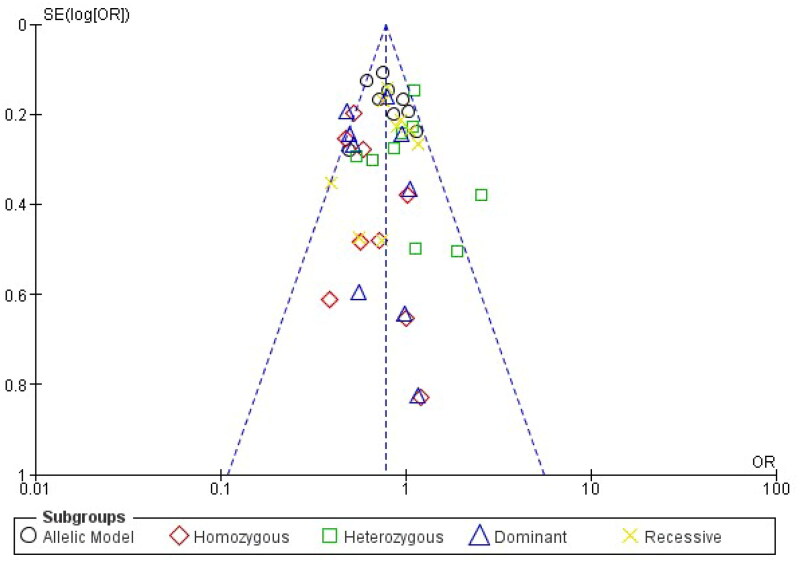
Funnel plot for publication bias.

Analysis of the rs1137101 polymorphism’s heterogeneity showed no significance within GG vs. AA (I2 = 0%), while moderate heterogeneity was distinguished with G vs. A (I2 = 38%), GG + GA vs. AA (I2 = 26%), GG vs GA + AA (I2 = 9%), GA vs AA (I2 = 47%) in the analyzed genetic models. Using the fixed effects model, which is based on heterogeneity (I2), T2DM has been showed to correlate substantially with genetic models for allelic OR = 0.79, (95%CI [0.70–0.88]), p= <0.00001, homozygote OR = 0.58, (95% CI [0.45–0.72]), p = <0.00001, dominant OR = 0.66, (95% CI [0.56–0.79]), p = <0.00001 and recessive OR = 0.83, (95% CI [0.71–0.96]), *p* = 0.01 genetic models. All of the data were displayed using forest plots. The sensitivity of the rs113710 study was imposed using Egger’s test, and Begg’s funnel plot presented with no publication bias for all five genetic models (Figures 3 and 4).

Publication bias arises when research with statistically significant results is more likely to be published, but studies with null or negative findings are less likely to be published. This bias creates significant issues for the accuracy of meta-analyses. In the context of the link between LEPR gene polymorphisms and T2DM, publication bias could lead to an overestimation of the genetic effect. To address this bias, a comprehensive approach is necessary. Firstly, conducting a funnel plot analysis can visually assess for asymmetry, where studies with larger sample sizes may have greater precision, potentially influencing the shape of the funnel. Secondly, quantitative methods like Egger’s test can quantify the extent of bias [31]. To further address publication bias, researchers can consider including grey literature and unpublished studies in their analysis, as these sources are less prone to selective reporting. Moreover, conducting a trim-and-fill analysis can impute missing studies, possibly lessening the effect of publication bias on the estimate of the overall effect size [41]. These methods collectively allow for a more rigorous exploration of publication bias and help ensure that the meta-analysis provides a more accurate representation of the true link between LEPR gene polymorphisms and the risk of T2DM.

## Discussion

T2DM, a multifaceted genetic condition closely linked to obesity, hypertension, gout, and lipid metabolism disorders, is commonly called insulin resistance (IR) syndrome in clinical contexts. It is described by compromised insulin functionality. Diabetes mellitus has surged worldwide, emerging as a widespread condition in recent decades. Recent data from the Global Burden of Disease 2019 highlights a substantial increase in the impact of non-communicable diseases (NCDs) on Disability Adjusted Life Years (DALYs), affecting both developed and developing nations over the past thirty years [42]. Recent studies suggest that individuals with IR may also resist the hormone leptin. Leptin, produced by adipocytes, is essential in regulating energy metabolism also maintaining lipid equilibrium with the body. It binds to the leptin receptor present within the brain and adipose tissue. Given its involvement in type 2 diabetes mellitus, the *LEPR* gene is frequently related to type 2 diabetes [43].

Case-control studies suggest that leptin modulates adipose tissue mass, and Certain LEPR polymorphisms may be associated with obesity-related disorders. Researchers recognized two polymorphisms: Lys109Arg, and Gln223Arg; there is a strong correlation between extracellular regions of the leptin receptor and conversion to type 2 diabetes in IGT patients with a higher risk. [44]. Numerous genetic association studies conducted by researchers have demonstrated the effectiveness of *LEPR* molecular variants in predicting T2D risk [45]. A polymorphism in this gene is established in different ethnic populations in these studies. However, the results of these studies were conflicting and inconclusive [46,47]. This case-control study suggests that when compared to controls, rs1137101 in the LEPR gene may influence the risk of T2DM. Further research will determine the precise relationship between *LEPR*-rs1137101 gene polymorphisms and T2DM within the study population. There is genetic variation in leptin levels across genotypes due to single nucleotide polymorphisms within the *LEPR* gene.

Based on a meta-analysis of 16 studies involving 7827 participants, evidence shows that the Gln223Arg polymorphism in *LEPR* is unrelated to vulnerability to type 2 diabetes. Based on the analysis stratified by ethnicity, no evidence was found for an association between polymorphism and type 2 diabetes in Caucasians or Asians [48]. The LEPR Pro1019Pro polymorphism is significantly linked to a greater chance of developing Type 2 Diabetes, according to a different meta-analysis study [49]. Genetic variants of *LEPR* are strongly linked to T2DM risk among Chinese, Asian, European, and Caucasian populations. According to a meta-analysis by Li et al. Chinese individuals with *LEPR* Gln223Arg polymorphisms through T2DM are more likely to develop the disease than those with other polymorphisms. A study’s results suggest that Chinese with the G allele of the *LEPR* Gln223Arg gene poly­morphism may have an increased risk of T2DM [18]. However, our findings have significant correlations between *LEPR* gene polymorphism and a higher risk of T2DM. Based on the meta-analysis findings, genetic variants of the *LEPR* gene may make people more prone to acquiring type 2 diabetes. These changes might affect the expression or function of the leptin receptor, a protein that regulates appetite and energy balance, perhaps paving the way for developing patient-specific treatment strategies.

The LEPR gene polymorphisms, such as Gln223Arg and rs1137101, have functional implications on leptin receptor activity, affecting its binding affinity and signal transduction, which play a crucial role in T2DM pathogenesis. These genetic variants can alter the structure and function of the leptin receptor, potentially leading to reduced sensitivity to leptin, a hormone crucial in regulating appetite, energy balance, and lipid metabolism. Recent research supports the functional implications of LEPR polymorphisms by highlighting their impact on leptin receptor activity and their link to T2DM risk. This information sheds light on the molecular mechanisms that connect genetic variations to the biological pathways implicated in the pathogenesis of T2DM [50]. The LEPR gene may be studied to determine whether mutations predispose individuals to leptin resistance. As obesity is a major risk factor for type 2 diabetes, genetic variations in leptin signalling mediators may have a role in the onset of the disease. To develop innovative therapy strategies aimed at enhancing leptin sensitivity by targeting variations in the LEPR gene. Novel and more potent approaches to the prevention and treatment of T2DM may result from a better understanding of the molecular and cellular mechanisms that modify leptin signalling. [51].

The risk factors for T2DM are both genetic and environmental. Understanding how these factors interact is necessary for developing prevention and treatment strategies. According to research, there is a strong hereditary component to T2DM among the different populations. This prevalence varies from 5% in relatively low-risk European communities to 20% in intermediate-risk minority groups of African and Hispanic descent in the United States. The disease has already been observed in 50% of Pima and South Sea Island communities. Therefore, genetic factors affecting type 2 diabetes risk may differ among ethnic groups [52,53]. Genetic association studies of type 2 diabetes should include a greater diversity of study populations. As longitudinal studies allow researchers to track changes over time in individuals, they can provide stronger evidence of causality than cross-sectional studies. In addition, it allows researchers to identify which LEPR gene variants are truly responsible for T2DM, and rule out confounding factors. In the future, researchers can use LEPR gene mutation studies to identify early risk factors, analyse the natural course of the disease, and verify causation.

Liu et al. and Su et al. conducted meta-analyses in 2015 and 2016, respectively, to examine the connection between the LEPR Gln223Arg gene variation and type 2 diabetes [48,49]. Both studies concluded that this gene variant did not influence T2DM susceptibility. However, these papers didn’t consider ethnic differences, with Liu’s analysis grouping all Asians into one category, and Su’s analysis combining Asians and Europeans. Additionally, some of the studies they included had control group genotypes that deviated from the expected Hardy-Weinberg Equilibrium (HWE), potentially contributing to differences in results [54]. These aspects enhance the credibility of our study’s results. However, our study has limitations and larger-scale studies are required to fully comprehend the relationship between the LEPR Gln223Arg gene polymorphism and T2DM. Additionally, it ignores additional variables that might influence plasma LEPR levels. [55].

Previous research supports the idea that the LEPR rs1137101 G > A polymorphism is linked to obesity and may predict variations in body composition [22]. Since obesity is a key factor in T2DM development, it’s suggested that LEPR signaling may be involved in T2DM’s origin. Numerous studies have recently examined the connection between the LEPR rs1137101 G > A polymorphism and T2DM risk, but the conclusions are still debatable. Numerous meta-analyses show that the LEPR rs1137101 G > A polymorphism and the risk of T2DM are not significantly correlated [32,48]. However, another meta-analysis argues that this polymorphism is a risk factor for T2DM [8]. We found a correlation between the LEPR rs1137101 G>A polymorphism and T2DM risk in our case-control study, but the ensuing meta-analysis failed to support this finding. More research is required to thoroughly investigate the association between the LEPR rs1137101 G>A polymorphism and T2DM risk in the future. Using replication studies across ethnic groups helps academics better understand the genetic links that are common to all populations. These data may assist in developing improved methods for preventing and treating illness.

## Conclusion

While numerous genes have been allied to an increased predisposition for emerging Type 2 Diabetes, the *LEPR* gene stands out as particularly significant, especially in specific populations. As the fields of genetics and diabetes research progress, we have an opportunity to gain a deeper understanding of how the *LEPR* gene, along with other genetic factors, impacts the risk of T2DM onset. Nevertheless, it remains imperative to conduct additional case-control studies involving substantial participant numbers to establish the validity and reliability of the previously observed associations with T2DM risk.

To achieve more precise and comprehensive results concerning the association between the *LEPR* gene and T2DM, a meta-analysis that consolidates the findings from multiple studies is warranted. Moreover, future investigations should encompass various confounding factors that could potentially influence the observed associations. By broadening the scope of research and incorporating larger sample sizes, researchers can further corroborate earlier discoveries and enrich our understanding of the role played by *LEPR* gene promoter polymorphisms in the susceptibility to T2DM.

## Data Availability

Not applicable. Data will not be disclosed due to patient confidentiality.
